# Cushing Syndrome in a Pediatric Patient with Topical Steroid Overuse

**DOI:** 10.1155/2022/8487737

**Published:** 2022-04-11

**Authors:** Sandip Kuikel, Sunil Aryal, Rupesh Shingh Basnyat, Suman Rimal

**Affiliations:** ^1^Shishuwa Hospital, 33700 Pokhara, Nepal; ^2^Department of Internal Medicine, Tribhuvan University Institute of Medicine, 44600 Kathmandu, Nepal

## Abstract

Cushing syndrome is a state of hypercortisolism from exogenous or endogenous exposure to glucocorticoids resulting in various clinical manifestations. In this case report, we present a case of a 15-month-old child who presented with cushingoid facies due to over-the-counter misuse of a very potent topical steroid (clobetasol 0.05%) for suspected scabies. Laboratory measurement of urinary free cortisol level was low, and 8 : 00 am basal cortisol level was measured, which was decreased, which confirmed the diagnosis of Cushing syndrome due to exogenous source. Over-the-counter topical steroids should not be used, and one should always consult a registered medical practitioner before using such products. Physicians when prescribing topical steroids should warn patients about the potential side effects of prolonged use of topical steroids.

## 1. Introduction

Cushing syndrome is a state of hypercortisolism from exogenous or endogenous exposure to glucocorticoids resulting in various clinical manifestations [1]. The most common manifestations of Cushing syndrome include obesity, moon face, hirsutism, facial plethora, muscle weakness, and glucose intolerance [2]. Causes of Cushing syndrome include exogenous administration of glucocorticoids, hypersecretion of adrenocorticotropic hormone (ACTH) from pituitary or other sources, and hypersecretion of cortisol from adenomas of the adrenal gland [3]. Cushing syndrome is a very rare condition in the pediatric age group, and its incidence is about 2–5 new cases per million per year [4]. Cushing syndrome due to exogenous administration of glucocorticoids is common; however, topical preparation resulting in Cushing syndrome is a rare phenomenon [5]. In this case report, we present the case of a 15-month-old child who presented with cushingoid facies due to over-the-counter misuse of a very potent topical steroid (clobetasol 0.05%) for suspected scabies.

## 2. Case Report

**Figure 1:**
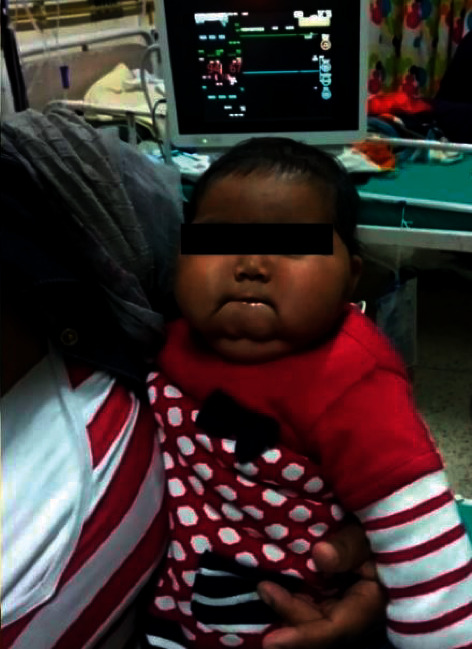
Moon facies of the child.

**Figure 2:**
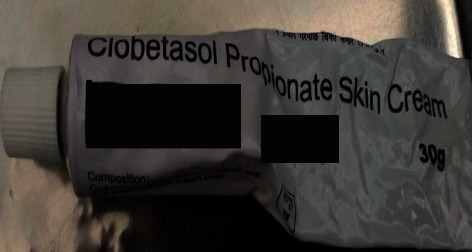
Very potent topical steroid used by the patient.

A 15-month-old child was brought to the outpatient department of our center with the chief complaint of swelling of the face with growth of excess facial hair. The mother also gives a history of excessive weight gain of the child evidenced by tightening of clothes and facial puffiness (Figure 1). The mother was breastfeeding the child and at the same time consumed prednisolone 5 mg once daily for the treatment as a maintenance dose of IgA nephropathy. The mother had been applying over-the-counter very potent topical steroid (clobetasol 0.05%) (Figure 2) for treatment of some dermatological conditions of the patient. The topical steroid was not prescribed, but was used as it used to relieve the symptoms of itching in the patient. The family history was suggestive of scabies as every member of the family had itchiness in the hands and groin region.

The child was born at home assisted by a midwife, and there were no complications at the time of delivery. The birth weight was not taken, but the mother states the child's birth weight was comparable to other newborns in her family. Developmental history was normal, and the growth of infants was comparable to others of the same age. There were no parental concerns regarding the growth and development of the child, but exact documentation of the child's height and weight had not been done.

On physical examination, the patient was 14 kg (above 97^th^ percentile) and the height of the patient was 40 cm (between 25^th^ and 50^th^ percentile). The patient had prominent facial hairs with moon facies. Blood pressure was raised for the corresponding height and age. There were no purple striae or facial plethora. Other system examinations were normal for the child's age. Routine blood tests were within the normal range including raised random blood sugar. The thyroid function test was normal.

In the background of the mother consuming prednisolone while breastfeeding and use of topical corticosteroids, the literature review was done which showed the safety of prednisolone in breastfeeding mothers, but there were reported cases of Cushing syndrome caused due to application of topical steroids for a long time. So, a provisional diagnosis of Cushing syndrome due to topical steroids was made. Laboratory measurement of 24-hour urinary free cortisol level was low; however, 8 : 00 am basal cortisol level was measured, which was decreased. This confirmed the diagnosis of Cushing syndrome due to an exogenous source of corticosteroids. Telemedicine consultation was done for the patient as the patient was too poor to afford treatment in the higher center. So, the patient was discharged with permethrin lotion for the treatment of scabies and discontinuation of topical steroids. Maintenance of steroid was done with 15 mg of oral prednisolone syrup in tapering doses over 45days.

On follow-up in 45 days, the moon face of the patient was persistent; however, local effects of topical steroids were reduced, and after three months, all the features of Cushing syndrome subsided. The patient is now on a regular follow-up to record the growth of the child. Recording of height for age, weight for age, and weight for height are being done and plotted in the World Health Organization (WHO) growth chart. This will help assess the residual effect, if any, of the erroneous use of steroids.

## 3. Discussion

Cushing syndrome is a rare condition in children; iatrogenic Cushing syndrome due to exogenous administration of steroids can be the cause, but topical steroid leading to Cushing syndrome is very unusual [6–9]. The recognition of Cushing syndrome by the treating physician is important for early diagnosis and treatment. Late-night salivary cortisol measurement, dexamethasone suppression test, and 24-hour urinary free cortisol determination are performed for the diagnosis of Cushing syndrome, followed by a stepwise evaluation to find out the cause [10]. Exogenous overuse of corticosteroids can be diagnosed based on classic signs of Cushing syndrome, and the confirmation of a decreased 8 : 00 am basal cortisol associated with low ATCH levels can be indicative of the diagnosis [3].

Siklar et al. described Cushing syndrome in a 9-month-old girl caused by long-term topical clobetasol propionate application. The patient was found to have severe adrenal suppression [6]. A similar phenomenon was described in a 72-year-old woman who developed manifestations of Cushing syndrome after long-term topical clobetasol propionate ointment for psoriasis. Shortly after tapering the dose of topical steroids, she developed signs of adrenal insufficiency (provoked by a urinary tract infection) requiring intravenous administration of hydrocortisone [7]. Ohnishi et al. [8], Ermis et al. [9], Al-Khenaizan et al. [11], Tiwari et al. [12], and Alkhuder et al. [13] described cases with a similar phenomenon to our case. Our patient had the use of a very potent topical steroid, and due to the presence of moon face with weight gain and laboratory finding of low 8 : 00 am basal cortisol, the diagnosis of Cushing syndrome due to exogenous steroid was confirmed. The family history of itching of the whole body more on axilla and groin suggested the diagnosis of scabies. Prolonged use of topical corticosteroids may cause Cushing syndrome, whereas suppression of the hypothalamic-pituitary-adrenal axis is less pronounced as reported in previous case reports [6–9]. The ACTH level and ACTH stimulation test as being normal rules out the suppression of HPA axis, thus not mandating the requirement of maintenance and tapering dose of steroid.

Topical steroids are frequently prescribed in the management of various dermatological conditions like dermatitis, lichen planus, psoriasis, pruritus ani, pemphigus, and vitiligo [14]. When used in these conditions, patients should be counseled about the potential side effects of topical steroids such as striae, purpura, telangiectasia, ulceration, easy bruising, rosacea, and hirsutism [15]. In a country like ours, where there is a misuse of topical steroids either by patients or by a medical practitioner, there can be a frequent occurrence of these potential side effects [16]. Though being rare, Cushing syndrome due to topical steroids in this situation can be observed as in our case.

When presented with the complaint of excessive weight gain, changes in a facial structure like moon face, excessive hair growth, and abdominal stria, Cushing syndrome should be suspected. It is often near impossible to extract the history of steroid use leading to Cushing syndrome in a setting like ours where most of the patients are just literate (able to read and write names only). So, in these cases, urinary free cortisol should be measured, and if the concentration is low, Cushing syndrome due to exogenous cause is suggested. Then, 8 : 00 am basal cortisol level should be measured, and if the level is low, the diagnosis of Cushing syndrome due to exogenous administration of glucocorticoids is confirmed [3]. Although the random blood sugar level was within normal range, the use of a glucose tolerance test is advised in such cases as the glucose metabolism is often disturbed in cases with excess corticosteroid resulting in hyperglycemia [17].

In our case, the topical steroid was not the drug of choice for the treatment of scabies but was permethrin. Inadvertent long-term use of topical steroids can cause thinning of the skin on the applied site, and steroids can permeate through this thinned out skin causing a systemic adverse effect like Cushing syndrome. Management of this kind of Cushing syndrome includes cessation of causative drugs and administration of corticosteroids in physiological dosage and gradually tapers it until the hypothalamic-pituitary-adrenal (HPA) axis normalizes [18]. In a low-resource setting like ours, the patency of the HPA axis could not be checked by the ACTH stimulation test, so assuming the axis was suppressed, we put the patient on an oral steroid for maintenance at 1 mg/kg dose tapering it over 45 days.

This manuscript reports a rare phenomenon of occurrence of Cushing syndrome with topical steroids. Some similar reported cases of Cushing syndrome due to exogenous topical steroid use can be found in a review of the literature. They provide gross ideas on the presentation, diagnosis, establishment of the integrity of the HPA axis, and treatment of such cases [6–9, 11–13]. However, early detection and management in a low-resource setting, with people just literate to read and write, is often difficult as described in this case. Because of limited resources, laboratory tests such as ACTH stimulation tests to evaluate the patency of the HPA axis could not be done in our case, and it is a limitation of this manuscript.

## 4. Conclusion

Topical steroids can cause various side effects. Over-the-counter topical steroids should not be used, and one should always consult a registered medical practitioner before using such products. Physicians when prescribing topical steroids should warn patients about the potential side effects of prolonged use of topical steroids. The patient should be advised to firmly apply topical steroids in thin layers and avoid exceeding the daily dose, and these preparations should only be used for a short course.

### 4.1. Patient Perspective

This manuscript draws the attention of patients toward the possible systemic side effects of potent topical steroids and the need for use as recommended by a registered practitioner only.

## Data Availability

The data used to support this study are included within the article.
