# Supplementary material for: Cushing Syndrome in a Pediatric Patient with Topical Steroid Overuse
Source: Case Rep Endocrinol. 2022 Apr 11;2022:8487737. doi: 10.1155/2022/8487737 (PMC9015878; doi:10.1155/2022/8487737)
Supplement: Supplementary Materials — CARE checklist is provided as a supplementary file. [file 8487737.f1.docx]

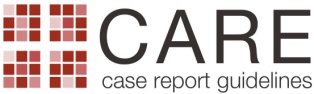
CARE Checklist of information to include when writing a case report
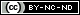


**Topic Item Checklist item description Reported on Line**

**Title 1** The diagnosis or intervention of primary focus followed by the words “case report” P-1

**Key Words 2** 2 to 5 key words that identify diagnoses or interventions in this case report, including "case report" P-1

Abstract

**(no references)**

**3a** Introduction: What is unique about this case and what does it add to the scientific literature? P-1

**3b** Main symptoms and/or important clinical findings P-1

**3c** The main diagnoses, therapeutic interventions, and outcomes P-1

**3d** Conclusion—What is the main “take-away” lesson(s) from this case? P-1

**Introduction 4** One or two paragraphs summarizing why this case is unique (**may include references**) P-1-2

**Patient Information 5a** De-identified patient specific information P2

**5b** Primary concerns and symptoms of the patient P2

**5c** Medical, family, and psycho-social history including relevant genetic information N/A

**5d** Relevant past interventions with outcomes P2

Clinical Findings

**Timeline**

**Diagnostic Assessment**

**Therapeutic Intervention**

**Follow-up and Outcomes**

1. Describe significant physical examination (PE) and important clinical findings P2
2. Historical and current information from this episode of care organized as a timeline N/A

**8a** Diagnostic testing (such as PE, laboratory testing, imaging, surveys). P2

**8b** Diagnostic challenges (such as access to testing, financial, or cultural) P2

**8c** Diagnosis (including other diagnoses considered) P2

**8d** Prognosis (such as staging in oncology) where applicable N/A

**9a** Types of therapeutic intervention (such as pharmacologic, surgical, preventive, self-care) P2

**9b** Administration of therapeutic intervention (such as dosage, strength, duration) P2

**9c** Changes in therapeutic intervention (with rationale) P2

**10a** Clinician and patient-assessed outcomes (if available) p2

**10b** Important follow-up diagnostic and other test results p2

**10c** Intervention adherence and tolerability (How was this assessed?) p2

**10d** Adverse and unanticipated events N/A

**Discussion 11a** A scientific discussion of the strengths AND limitations associated with this case report P4

**11b** Discussion of the relevant medical literature **with references** P3-4

**11c** The scientific rationale for any conclusions (including assessment of possible causes) P4

**11d** The primary “take-away” lessons of this case report (without references) in a one paragraph conclusion P4

**Patient Perspective 12** The patient should share their perspective in one to two paragraphs on the treatment(s) they received p4

**Informed Consent 13** Did the patient give informed consent? Please provide if requested . . . . . . . . . . . . . . . . . . . . . . . . . . . . . . . . . . . . . . **Yes √ No**
